# Impact and usefulness of the transition to the new MAFLD classification for non-B, non-C HCC: a retrospective cohort study

**DOI:** 10.1186/s12876-023-02851-y

**Published:** 2023-06-28

**Authors:** Yusuke Johira, Takashi Nakahara, Takahiro Kinami, Shintaro Yamasaki, Masanari Kosaka, Yuki Shirane, Ryoichi Miura, Serami Murakami, Shigeki Yano, Kei Amioka, Kensuke Naruto, Yuwa Ando, Yumi Kosaka, Kenichiro Kodama, Shinsuke Uchikawa, Hatsue Fujino, Atsushi Ono, Eisuke Murakami, Wataru Okamoto, Masami Yamauchi, Tomokazu Kawaoka, C. Nelson Hayes, Masataka Tsuge, Michio Imamura, Hiroshi Aikata, Shiro Oka

**Affiliations:** Department of Gastroenterology, Graduate School of Biomedical and Health Sciences, Hiroshima University, Hiroshima, Japan

**Keywords:** Metabolic dysfunction-associated fatty liver disease (MAFLD), Nonalcoholic fatty liver disease (NAFLD), Hepatocellular carcinoma (HCC)

## Abstract

**Background:**

Metabolic dysfunction-associated fatty liver disease (MAFLD) represents a new classification system for fatty liver disease. In this study, we investigated the clinical characteristics of patients with MAFLD-hepatocellular carcinoma (HCC) in comparison with those with nonalcoholic fatty liver disease (NAFLD) and considered the validity and challenges of the new criteria.

**Methods:**

This study included 237 untreated non-B, non-C HCC patients with hepatic steatosis. We examined the profile and laboratory findings of patients with MAFLD-HCC and NAFLD-HCC. We also classified MAFLD-HCC patients according to the factors on which the diagnosis was based and compared their clinical characteristics.

**Results:**

A total of 222 (94%) and 101 (43%) patients were diagnosed with MAFLD and NAFLD, respectively. MAFLD-HCC patients were more likely to be male than NAFLD-HCC, but there were no significant differences in metabolic indices, noninvasive liver fibrosis score or HCC status. In a study of MAFLD-HCC patients by diagnostic factor, those with overweight only were younger and had advanced liver fibrosis histologically, and when limited to patients younger than 70 years, the majority were overweight. Redefinition of overweight as BMI ≥ 25 reduced the number of MAFLD-HCC patients by only 5, from 222 to 217.

**Conclusions:**

MAFLD accounted for the majority of non-B, non-C HCC cases with hepatic steatosis. Examination of additional cases and revision of the detailed criteria is needed so that it can be used to efficiently select patients with fatty liver who are at high risk of developing HCC.

**Supplementary Information:**

The online version contains supplementary material available at 10.1186/s12876-023-02851-y.

## Introduction

Nonalcoholic fatty liver disease (NAFLD) is one of the most common chronic liver diseases, estimated to affect about 25% of the world population [1–3]. It is a progressive condition leading not only to fatal liver-related outcomes including cirrhosis, liver cancer, and liver failure, but also to extrahepatic complications [4–7] such as cardiovascular disease [8, 9], chronic kidney disease [10, 11], and extra-hepatic cancers [12, 13]. Increasing prevalence of NAFLD is an urgent medical and public health issue to be addressed [14, 15].

The term NAFLD was first coined by Jurgen Ludwig et al. in 1980 [16]. Now it is defined as imaging or pathogenic evidence of hepatic steatosis without any secondary factors leading to hepatic fat accumulation, such as excessive alcohol intake and long-term use of steatogenic medication [17]. Previous studies have shown that many factors are involved in the development and progression of NAFLD, including insulin resistance [18, 19], genetic factors [20, 21], oxidative stress [22, 23] and the gut microbiome [24, 25], which is called the “multiple parallel hits hypothesis [26]”.

On the other hand, the inaccuracy and divergence from clinical practice of the term NAFLD have long been noted [27, 28]. Thus, metabolic dysfunction-associated fatty liver disease (MAFLD) was proposed as a comprehensive concept to replace NAFLD [29]. MAFLD is diagnosed in patients with one or more of the following conditions in addition to hepatic steatosis: overweight, type 2 diabetes mellitus (DM), and metabolic risk abnormalities (MRA) in lean or normal weight patients [30]. MAFLD criteria do not require the exclusion of other causes of liver disease such as excessive alcohol consumption or viral hepatitis like those of NAFLD, which is an advantage in terms of consistency with the clinical situation of patients. Moreover, in a large cohort study of individuals enrolled in the third National Health and Nutrition Examination Surveys (NHANES III), patients diagnosed with MAFLD were older, had higher body mass index (BMI), incidence of metabolic complications including type 2 DM and hypertension, and presence of non-invasive biomarkers of liver fibrosis than NAFLD patients, which suggested that MAFLD criteria could identify patients at higher risk of intrahepatic or extrahepatic complications [31]. However, there is also a concern that renaming NAFLD to MAFLD can exclude lean or normal weight patients who do not have metabolic abnormalities but still have a high risk of developing intrahepatic or extrahepatic diseases due to severe hepatic steatosis, as the MAFLD diagnostic criteria do not consider the severity of steatosis [32].

Thus, the superiority of MAFLD to NAFLD is still unclear, and further studies are needed. Furthermore, there is an overwhelming lack of studies comparing the two criteria in patients with hepatocellular carcinoma (HCC). HCC is a poor prognosis complication of fatty liver disease, and is not only due to viral or alcoholic hepatitis [33] as in the past, but also metabolic abnormalities such as recently increasing obesity or type 2 DM [34]. In this study, we investigated the clinical characteristics of MAFLD patients developing HCC in comparison with those with NAFLD, and considered the validity and challenges of the new criteria.

## Methods

### Study population

**Fig. 1:**
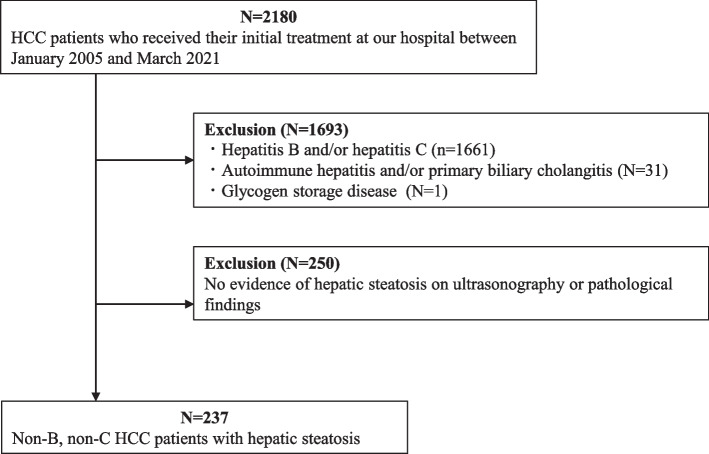
Patient selection and study design. From the 2,180 reviewed patients with HCC before their initial treatment, we excluded those diagnosed with viral hepatitis, autoimmune hepatitis, primary biliary cholangitis, and glycogen storage disease, and selected 237 patients with hepatic steatosis determined by pathological or ultrasonography findings

We reviewed 2,180 HCC patients for whom sufficient information was available with respect to physical and laboratory findings, alcohol consumption and medication, and who received their initial treatment at Hiroshima University Hospital between January 2005 and March 2021. We excluded patients diagnosed with hepatitis B and/or hepatitis C (*n* = 1,661), autoimmune hepatitis and/or primary biliary cholangitis (*n* = 31), or glycogen storage disease (*n* = 1) (Fig. 1). The criteria for hepatitis B were positive hepatitis B surface antigen or history of antiviral treatment, and those of hepatitis C were positive anti hepatitis C virus antibody. Among the 487 patients who remained after applying exclusion criteria, 237 patients were enrolled in the study who had or had previously had hepatic steatosis on ultrasonography or pathological findings. All of them were Japanese. In addition, excluded hepatitis C cases were compared with the target cases in some studies.

### Diagnostic criteria

All patients in this study were screened for the criteria for MAFLD and NAFLD based on their physical and laboratory findings prior to treatment for HCC. Hepatic steatosis was determined pathologically by fatty accumulation in more than 5% of the hepatocytes in a specimen or by the presence of findings on ultrasonography, i.e., increased hepatic echogenicity, intrahepatic vascular blurring, ultrasound attenuation or controlled attenuation parameter value of 230 dB/m or higher. Diagnostic criteria for MAFLD were largely in accordance with those proposed by the international expert consensus statement [30] regarding the presence of one or more of the three conditions (overweight, type 2 DM, and MRA in lean or normal weight patients). Overweight was defined as BMI ≥ 23 kg/m^2^. The diagnosis of type 2 DM was based on diagnostic and treatment history or laboratory findings; either fasting blood glucose (FBS) ≥ 126 mg/dl, 75 g oral glucose tolerance test (OGTT) 2-h value ≥ 200 mg/dl, or casual blood glucose ≥ 200 mg/dl plus glycated hemoglobin (HbA1c) ≥ 6.5%. MRA included the following six items, and MAFLD was diagnosed in lean or normal weight patients if two or more of them were met; (a) waist circumference ≥ 90 cm in men and 88 cm in women, (b) blood pressure ≥ 130/85 mmHg or on specific drug treatment, (c) plasma triglycerides (TG) ≥ 150 mg/dl or on specific drug treatment, (d) plasma high-density lipoprotein cholesterol (HDL-C) < 40 mg/dl for men and 50 mg/dl for women or on specific drug treatment, (e) prediabetes (FBS 100 to 125 mg/dl or HbA1c 5.7 to 6.4%), (f) homeostasis model assessment of insulin resistance (HOMA-IR) score ≥ 2.5. Although the original criteria for MRA included an item referring to C-reactive protein (CRP) level, we did not include it since CRP levels of the patients in this study were considered to be affected by the status of HCC. NAFLD was diagnosed in patients without habitual drinking or light drinkers (defined as less than 30 g of ethanol equivalent per day for men and 20 g for women).

### Variables

Clinical background measurements included the year of HCC diagnosis, age, gender, BMI, waist circumference, alcohol consumption and incidence of type 2 DM, hypertension, and dyslipidemia. Regarding alcohol consumption, all patients were classified into four categories: none, light, moderate, and heavy drinkers. Those with no habitual drinking were classified as “none”. The definition of light drinkers is described above. Heavy drinkers were defined as those who drank 60 g of ethanol equivalent or more per day, and moderate drinkers as those who drank between light and heavy drinkers. Hypertension was defined as blood pressure ≥ 130/85 mmHg or on specific drug treatment. Dyslipidemia was defined as HDL-C < 40 mg/dl for men and 50 mg/dl for women or TG ≥ 150 mg/dl or on specific drug treatment. Laboratory measurements included platelet count, prothrombin activity (PT), total bilirubin (T-Bil), aspartate aminotransferase (AST), alanine aminotransferase (ALT), γ-glutamyl transferase (γ-GTP), alkaline phosphatase (ALP), lactate dehydrogenase (LDH), choline esterase (ChE), albumin (ALB), ammonia, TG, HDL-C, low-density lipoprotein cholesterol (LDL-C), FBS, HbA1c, alpha-fetoprotein (AFP), and des-γ-carboxy prothrombin (DCP). Non-invasive liver fibrosis assessment included fibrosis-4 index (Fib-4 index) and NAFLD fibrosis score (NFS). The number and maximum diameter of tumors and clinical stage assessed by general rules for the clinical and pathological study of primary liver cancer 6^th^ edition were listed as indicators of HCC progression. All of these data were taken before the initial treatment for HCC.

### Statistical analysis

Continuous variables were expressed as median with range. Categorical variables were expressed as total numbers and percentages. Differences between the groups were investigated using Fisher’s exact test for categorial variables and Mann–Whitney U test or Kruskal–Wallis test for continuous variables. Results with a *p* value < 0.05 were considered to be statistically significant. All analysis was conducted using EZR 4.0.3.

## Results

### Patient profile

Of the 237 patients enrolled in this study, 198 (84%) were male, with a median age of 73 years and a median BMI level of 24.9 (Table 1). The median Fib-4 index and NFS were 3.23 and 0.67, respectively. 135 (57%) patients had type 2 DM, 157 (66%) had hypertension, and 118 (50%) had dyslipidemia. They were older, had higher BMI and prevalence of type 2 DM, hypertension and dyslipidemia than hepatitis C patients excluded during the case selection. While there was no significant difference in NFS, Fib-4 index was significantly lower in the target population.

**Table 1:** Clinical characteristics of non-B, non-C HCC patients with hepatic steatosis and HCV-HCC patients

Variables	non-B, non-C HCC (*n* = 237)	HCV-HCC (*n* = 1154)	*P* value
Age (years)	73 (30–90)	71 (28–96)	< 0.001
Male (%)	198 (84)	780 (68)	< 0.001
BMI (kg/m^2^)	24.9 (15.2–45.9)	22.5 (13.4–38.9)	< 0.001
Type 2 diabetes (%)	135 (57)	311 (27)	< 0.001
Hypertension (%)	157 (66)	519 (45)	< 0.001
Dyslipidemia (%)	118 (50)	360 (32)	< 0.001
Fib-4 index	3.23 (0.51–14.6)	5.03 (0.65–51.5)	< 0.001
NFS	0.67 (-4.99 – 3.89)	0.78 (-5.60 – 18.9)	0.368

Data are expressed as medians with ranges for continuous variables and as total numbers and percentages for categorical variables. Differences between the groups are investigated using Fisher’s exact test for categorial variables or Mann–Whitney U test for continuous variables

**Fig. 2:**
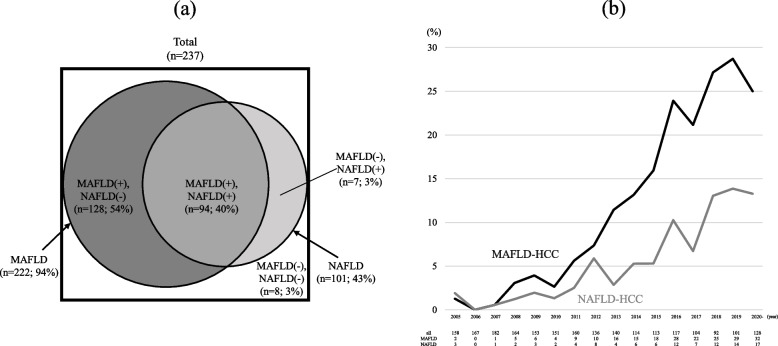
**a** MAFLD and NAFLD patients in 237 cases of non-B, non-C HCC with hepatic steatosis. 222 (94%) and 101 (43%) patients were diagnosed with MAFLD and NAFLD, respectively. 94 (40%) patients met both of the two criteria, 128 (54%) met only MAFLD criteria, 7 (3%) met only NAFLD criteria, and 8 (3%) met neither. **b** Trends in the proportion of MAFLD and NAFLD patients among all HCC cases. Both have been on the rise, with MAFLD-HCC in particular increasing significantly, accounting for around 25% of the total cases. The difference between MAFLD-HCC and NAFLD-HCC is larger than before

Of the 237 non-B, non-C HCC patients with hepatic steatosis, 222 (94%) and 101 (43%) were diagnosed with MAFLD and NAFLD, respectively; 94 (40%) met both of the two criteria, 128 (54%) met only MAFLD, 7 (3%) met only NAFLD (Fig. 2a). There were 8 (3%) patients who were not diagnosed with either MAFLD or NAFLD. Table S1 shows alcohol consumption for each group. In the MAFLD (+) / NAFLD (-) group, 73 (57%) and 55 (43%) patients were moderate and heavy drinkers, respectively. In the MAFLD (+) / NAFLD (+) group, 14 (15%) patients were light drinkers and the remaining 80 (85%) had no drinking habits.

### Clinical characteristics of MAFLD-HCC patients

Compared with non-MAFLD-HCC patients, MAFLD-HCC patients had higher prevalence of type 2 DM and dyslipidemia, higher BMI, ChE and ammonia, and worse metabolic indices; higher TG, FBS, HbA1c, HOMA-IR, and lower HDL-C (Table 2). NFS was higher in MAFLD-HCC patients than in non-MAFLD-HCC patients (0.75 vs -1.43), and Fib-4 index tended to be higher in MAFLD-HCC patients while there was no significant difference.

**Table 2:** Clinical characteristics of MAFLD and NAFLD patients

						***P* value**		
**Variables**	**Total (*n* = 237)**	**MAFLD (*n* = 222)**	**non-MAFLD (*n* = 15)**	**NAFLD** **(*n* = 101)**	**non-NAFLD (*n* = 136)**	**MAFLD vs non-MAFLD**	**NAFLD vs non-NAFLD**	**MAFLD vs NAFLD**
Age (years)	73 (30–90)	73 (30–90)	75 (48–90)	76 (48–90)	72 (30–90)	0.733	0.072	0.24
Male (%)	198 (84)	186 (84)	12 (80)	67 (66)	131 (96)	0.719	< 0.001	< 0.001
BMI (kg/m^2^)	24.9 (15.2–45.9)	25.1 (15.3–45.9)	20.8 (15.2–23.0)	25.1 (15.3–45.9)	24.3 (15.2–38.4)	< 0.001	0.188	0.941
Waist circumference (cm)	84.9 (58.5–125.7)	85.5 (58.5–125.7)	77.8 (74–82.9)	87.4 (66.8–125.7)	82.6(58.5–100.1)	0.126	0.068	0.276
Type 2 diabetes (%)	135 (57)	135 (61)	0	59 (58)	76 (56)	< 0.001	0.791	0.714
Hypertension (%)	157 (66)	150 (68)	7 (47)	66 (65)	91 (67)	0.155	0.890	0.704
Dyslipidemia (%)	118 (50)	118 (53)	0	54 (53)	64 (47)	< 0.001	0.359	1
Platelet count (× 10^4^/μL)	16.0 (3.4–57.3)	15.7 (3.4–57.3)	19.4 (11.6–30.7)	16.2 (3.4–45.6)	16.0 (4.6–57.3)	0.013	0.860	0.817
PT (%)	86 (8.1–128)	86 (8.1–128)	79 (10.5–123)	89 (10.5–123)	86 (8.1–128)	0.388	0.119	0.393
T-Bil (mg/dL)	0.8 (0.2–7.7)	0.8 (0.2–7.7)	0.8 (0.4–2.8)	0.8 (0.3–7.7)	0.8 (0.2–6.2)	0.897	0.459	0.654
AST (U/L)	38 (14–293)	38 (14–220)	42 (18–293)	38 (17–293)	38 (14–220)	0.949	0.778	0.859
ALT (U/L)	33 (9–462)	33 (9–278)	26 (10–462)	34 (11–269)	33 (9–462)	0.528	0.453	0.703
γ-GTP (U/L)	85 (14–1431)	85 (14–1431)	63 (20–945)	69 (14–1431)	104 (19–879)	0.254	0.005	0.055
ALP (U/L)	321 (95–1503)	322 (95–1503)	293 (146–1082)	329 (96–1082)	310 (95–1503)	0.224	0.937	0.901
LDH (U/L)	204 (87–920)	206 (87–611)	204 (131–920)	214 (87–504)	201 (95–920)	0.941	0.038	0.178
ChE (U/L)	232 (51–473)	238 (51–473)	180 (93–305)	244 (72–431)	224 (51–473)	0.013	0.004	0.139
ALB (g/dL)	3.9 (1.9–5.2)	4.0 (1.9–5.0)	3.9 (2.8–5.2)	4.0 (2.6–5.0)	3.9 (1.9–5.2)	0.66	0.057	0.202
Ammonia (μg/dL)	39 (10–136)	40 (10–136)	29 (17–91)	37 (10–136)	39 (10–108)	0.011	0.536	0.465
TG (mg/dL)	100 (33–321)	101 (33–321)	69 (34–131)	101 (34–291)	98 (33–321)	0.002	0.619	0.905
HDL-C (mg/dL)	48 (12–100)	47 (12–100)	56 (42–73)	47 (12–96)	49 (22–100)	0.033	0.587	0.948
LDL-C (mg/dL)	98 (15–382)	98 (15–382)	89 (46–115)	94 (15–189)	98 (19–382)	0.245	0.409	0.505
FBS (mg/dL)	111 (70–299)	113 (70–299)	93 (72–147)	112 (76–299)	110 (70–270)	< 0.001	0.103	0.67
HbA1c (%)	6.1 (3.6–13.1)	6.2 (3.6–13.1)	5.2 (4.2–5.6)	6.2 (3.9–13.1)	6.1 (3.6–13.1)	< 0.001	0.493	0.799
HOMA-IR	3.93 (0.11–45.0)	4.07 (0.11–45.0)	1.49 (0.36–13.2)	4.45 (0.11–45.0)	3.51 (0.36–23.2)	0.003	0.049	0.355
Fib-4 index	3.23 (0.51–14.6)	3.33 (0.51–14.6)	2.32 (0.67–12.6)	3.42 (0.51–14.6)	3.19 (0.63–11.4)	0.115	0.681	0.957
NFS	0.67 (-4.99 – 3.89)	0.75 (-4.99 – 3.89)	-1.43 (-4.32 –1.31)	0.81 (-4.99 – 3.46)	0.54 (-4.25 – 3.89)	< 0.001	0.584	0.882

Data are expressed as medians with ranges for continuous variables and as total numbers and percentages for categorical variables. Differences between the groups are investigated using Fisher’s exact test for categorial variables or Mann–Whitney U test for continuous variables

*BMI* body mass index, *PT* prothrombin activity, *T-Bil* total bilirubin, *AST* aspartate aminotransferase, *ALT* alanine aminotransferase, *γ-GTP* γ-glutamyl transferase, *ALP* alkaline phosphatase, *LDH* lactate dehydrogenase, *ChE* choline esterase, *ALB* albumin, *TG* plasma triglycerides, *HDL-C* high-density lipoprotein cholesterol, *LDL-C* low-density lipoprotein cholesterol, *FBS* fasting blood glucose, *HbA1c* glycated hemoglobin, *HOMA-IR* homeostasis model assessment of insulin resistance, *Fib-4 index* fibrosis-4 index, *NFS* NAFLD fibrosis score

### Clinical characteristics of NAFLD-HCC patients

Compared with non-NAFLD-HCC patients, NAFLD-HCC patients were more likely to be female, had lower γ-GTP and higher LDH, ChE, and HOMA-IR levels (Table 2). There were no significant differences in the age, non-invasive liver fibrosis scores, and the prevalence of metabolic complications including type 2 DM, hypertension, and dyslipidemia between the two groups.

### Comparison between MAFLD-HCC and NAFLD-HCC patients

In a comparison between MAFLD-HCC and NAFLD-HCC groups, gender composition was the only item which showed a significant difference; MAFLD-HCC patients were more likely to be male (Table 2). Age, prevalence of metabolic complications, laboratory findings, and non-invasive liver fibrosis scores were not statistically different.

### HCC associated factors

Table 3 shows tumor-related factors for MAFLD-HCC, NAFLD-HCC and hepatitis C-HCC. There were no significant differences between MAFLD-HCC and NAFLD-HCC in AFP and DCP level, the number of tumors, the maximum tumor size, or clinical stage. On the other hand, both of them were larger and therefore in more advanced clinical stage than HCC induced by hepatitis C.

**Table 3:** Comparison of HCC associated factors in patients with MAFLD, NAFLD, and hepatitis C

					***P* value**	
**Variables**	**HCV (*n* = 1154)**	**MAFLD (*n* = 222)**	**NAFLD (*n* = 101)**	**HCV vs MAFLD**	**HCV vs NAFLD**	**MAFLD vs NAFLD**
AFP (ng/mL)	20.7 (0.5–538,100)	8.8 (1.0–1,089,700)	9.5 (1.0–173,820)	< 0.001	0.001	0.996
DCP (mAU/mL)	63 (3.3–1,160,000)	163 (11–593,860)	235 (13–593,860)	< 0.001	< 0.001	0.73
Number of tumors						
1	609 (53)	118 (53)	58 (57)	0.942	0.406	0.547
2,3	294 (25)	53 (24)	24 (25)	0.673	0.811	1
4–9	95 (8)	18 (8)	6 (6)	1	0.566	0.648
10-	156 (14)	33 (15)	13 (13)	0.595	1	0.732
Maximum tumor size (mm)						
< 20	407 (35)	38 (17)	14 (14)	< 0.001	< 0.001	0.516
20–100	710(62)	164 (74)	76 (75)	< 0.001	0.007	0.891
100 <	37 (3)	20 (9)	11 (11)	< 0.001	< 0.001	0.684
Clinical stage						
Stage II ≤	848 (73)	194 (87)	86 (85)	< 0.001	0.009	0.599
Stage III ≤	455 (39)	109 (49)	49 (49)	0.009	0.09	1
Stage IV ≤	176 (15)	40 (18)	16 (16)	0.314	0.89	0.752

Data are expressed as medians with ranges for continuous variables and as total numbers and percentages for categorical variables. Differences between the groups are investigated using Fisher’s exact test for categorial variables or Mann–Whitney U test for continuous variables

*AFP* alpha-fetoprotein, *DCP* des-γ-carboxy prothrombin

### Trends in the proportion of MAFLD-HCC and NAFLD-HCC patients

Figure 2b shows the trends in the proportion of MAFLD-HCC and NAFLD-HCC patients including those who met both diagnostic criteria among all of the reviewed HCC individuals (*n* = 2180) by the year of diagnosis. The number of all HCC patients diagnosed in the year and MAFLD-HCC and NAFLD-HCC patients among them are shown at the bottom. While the proportions of both were less than 5% prior to 2010, they have been on the rise with MAFLD-HCC in particular increasing significantly, accounting for around 25% of the total cases in recent years. NAFLD-HCC, on the other hand, has increased to just under 15%, and the difference between the two is larger than before.

### Categorization of MAFLD-HCC patients

**Fig. 3:**
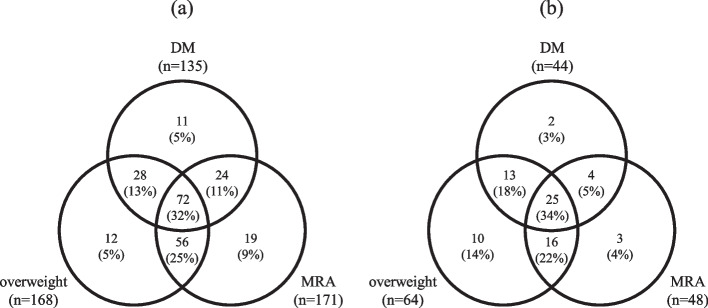
**a** Proportion of diagnostic factors in MAFLD patients. 11 (5%), 12 (5%) and 19 (9%) patients had only DM, overweight and MRA, respectively, and 28 (13%) had DM and overweight, 24 (11%) had DM and MRA, 56 (25%) had overweight and MRA, and 72 (32%) had all three components for the diagnosis of MAFLD. **b** Proportion of diagnostic factors in MAFLD patients under 70 years of age. 2 (3%), 10 (14%) and 3 (4%) patients had only DM, overweight and MRA, respectively. 13 (18%) had DM and overweight, 4 (5%) had DM and MRA, 16 (22%) had overweight and MRA, and 25 (34%) had all three

**Fig. 4:**
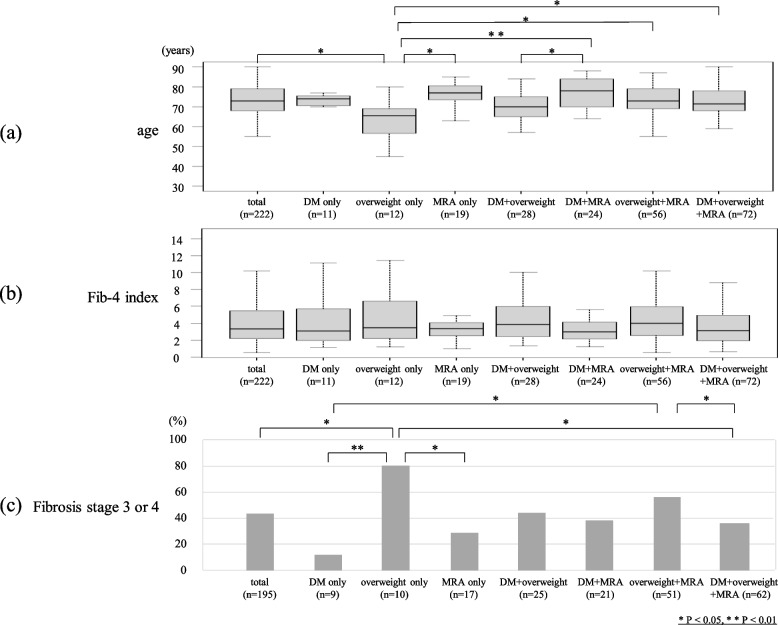
**a** Age in each group. The patients with only overweight were younger than the overall population, those in the MRA-only group, DM + MRA group, overweight + MRA group, and the group with all three factors. **b** Fib-4 index in each group. There was no significant difference between the groups. **c** The ratio of cases with histological fibrosis stage 3 or 4 in each group. The percentage was higher in the overweight-only group (80%) than the overall population (44%), DM-only group (11%), MRA-only group (29%), and the group with all three factors (37%)

Figure 3a shows the proportion of the factors on which the diagnosis was based in MAFLD-HCC patients (*n* = 222). The number of patients with DM, overweight, and MRA were 135 (61%), 168 (76%) and 171 (77%), respectively. 11 (5%), 12 (5%) and 19 (9%) patients had only DM, overweight and MRA, respectively, and 28 (13%) had DM and overweight, 24 (11%) had DM and MRA, 56 (25%) had overweight and MRA, and 72 (32%) had all three of the components for the diagnosis of MAFLD. Figure 4a shows an age comparison of the groups: the median age of the patients with only overweight was 65 years, significantly younger than the overall population (73 years) and also younger than MRA-only group (77 years), DM + MRA group (78 years), overweight + MRA group (73 years), and the group with all three (72 years). As shown in the Fib-4 index comparison in Fig. 4b, there was no significant difference between the groups. In the study of histologically evaluable individuals (*n* = 195), the percentage of advanced liver fibrosis cases with fibrosis stage 3 or 4 was 80% in overweight-only group, higher than the overall population (44%), DM-only group (11%), MRA-only group (29%), and the group with all three (37%) (Fig. 4c). Figure 3b shows the proportion of the diagnostic factors in MAFLD-HCC patients younger than 70 years (*n* = 73). 2 (3%), 10 (14%) and 3 (4%) patients had only DM, overweight and MRA, respectively. 13 (18%) had DM and overweight, 4 (5%) had DM and MRA, 16 (22%) had overweight and MRA, and 25 (34%) had all of the three.

### Impact of redefining overweight

**Fig. 5:**
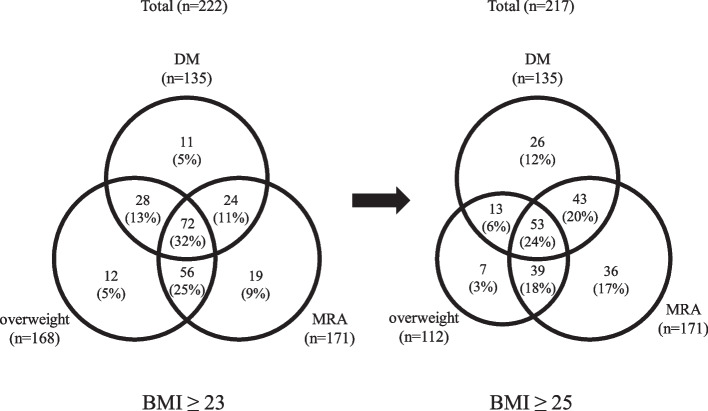
Change in the proportion of diagnostic factors of MAFLD patients due to redefinition of overweight. Although the number of overweight patients decreased by 56, from 168 to 112, most of them still met the MAFLD criteria due to DM and/or MRA comorbidity; only 5 patients were omitted

Figure 5 shows the change in the distribution of diagnostic factors when overweight is redefined as BMI ≥ 25 kg/m^2^. Although the number of overweight patients decreased by 56, from 168 to 112, most of them still met the MAFLD criteria due to DM and/or MRA comorbidity; only 5 patients were omitted.

## Discussion

We examined the clinical characteristics of non-B, non-C HCC cases with hepatic steatosis and considered the validity of MAFLD. MAFLD is a new classification for fatty liver disease that focuses on the presence of metabolic dysfunction. There have been many reports published on the relationship between hepatic carcinogenesis and metabolic dysfunction. Accumulated visceral fat and type 2 DM contribute to the development of HCC through various mechanisms, including inflammatory cytokines, several endocrine pathways, insulin resistance, hepatic lipotoxicity, hepatic and peripheral insulin resistance, and oxidative stress [35–37].

Although MAFLD criteria originally allow for viral hepatitis complication, in this study we focused on HCC with fatty liver disease associated with metabolic abnormalities as the primary cause, and excluded viral HCC cases. Actually, the target population was characterized by complication of metabolic dysfunction with a higher incidence of lifestyle-related diseases than the excluded hepatitis C cases. The difference in tumor progression at diagnosis between the target cases and the hepatitis C cases shown in Table 3 may be due to the difference in regular screening; hepatitis C patients might be followed more carefully leading to early detection of HCC [38]. In recent years, fatty liver disease has become an increasingly important cause of chronic liver disease and consequent HCC, and it is an urgent issue to identify cases with high risk of carcinogenesis.

MAFLD is expected to include cases with higher risk of intrahepatic or extrahepatic complications than the conventional disease concept of NAFLD [39–42]. The prevalence of MAFLD and NAFLD calculated in general population were nearly equivalent, 33.2% and 31.2% respectively, with 28.6% overlap [31]. However, the composition in our study, which included only patients with HCC, was quite different, and characterized by a large proportion of patients who met only MAFLD criteria **(**Fig. 2a). It should be emphasized that MAFLD-HCC accounted for the majority of the total cases, and was clearly more common than NAFLD-HCC. Heavy drinkers were not excluded in this study since they are more likely to suffer from obesity and metabolic complications. Therefore, there were 55 patients who drank more than 60 g of ethanol equivalent per day among the 128 patients who met only MAFLD criteria (Table S1). In other words, the remaining 73 patients were encompassed only by MAFLD criteria not by alcoholic hepatitis or NAFLD. In addition, it is expected that there were a certain number of patients who had lost the findings of hepatic steatosis due to the progression of hepatic fibrosis among the 250 patients excluded because of the absence of the findings, and they might have been previously diagnosed as MAFLD or NAFLD [43]. Therefore, we applied their physical and laboratory findings to the criteria for MAFLD and NAFLD other than the presence of fatty liver, and found that a total of 222 (89%) and 91 (36%) cases met the alternative criteria, respectively, which was similar to the results we obtained in the original target subjects. MAFLD cases accounted for a large part of non-B, non-C HCC cases, suggesting the usefulness of the disease concept as a new definition for following patients with fatty liver at high risk of carcinogenesis.

Both the decline in the number of new patients with viral HCC due to the development of antiviral therapy and the increase in the number of patients with metabolic syndrome have led to an increase in the proportion of fatty liver disease among all causes of HCC [44, 45]. The trends in the proportion of MAFLD-HCC and NAFLD-HCC patients we showed in Fig. 2b were consistent with these findings. It is also noteworthy that MAFLD-HCC was more common than NAFLD-HCC in most of the years in our study period, and that the difference has been larger than before. These results illustrate the validity of the disease concept of MAFLD, as an increasing number of patients meet only the criteria for MAFLD, in other words, develop HCC due to moderate or heavy drinking habits or some metabolic disorder.

On the other hand, we should also focus on the 7 patients who met only the diagnostic criteria for NAFLD (Fig. 2a). They have developed HCC, even though they were non-obese patients without excessive alcohol intake, had no metabolic complications except for one patient with hypertension, and had non-invasive liver fibrosis scores with a median Fib-4 index value of 1.80 and a median NFS value of -2.03, which were significantly lower than those of the overall population. In other words, there might have been a carcinogenic risk that could not be accounted for by considering metabolic dysfunction alone. In addition to environmental factors such as obesity and lifestyle-related diseases, genetic factors such as patatine-like phospholipase domain containing 3 (PNPLA3) single nucleotide polymorphism [46, 47] are generally known to be involved in the development and progression of NAFLD, which may have contributed to their carcinogenesis. However, the role of genetic factors in the pathogenesis, development and progression of MAFLD has not yet been fully elucidated, and this remains a challenge for the future.

MAFLD-HCC patients had worse metabolic indices for carbohydrates and lipids than non-MAFLD-HCC patients, which reflects the disease concept of MAFLD focusing on metabolic dysfunction. On the other hand, there were no significant differences in metabolic indices other than insulin resistance between NAFLD-HCC and non-NAFLD-HCC patients. The difference between the two groups was in the amount of alcohol consumed, and the smaller proportion of men and lower levels of γ-GTP in the NAFLD-HCC group may reflect the fact that alcoholic liver injury is more common among men in Japan.

Although MAFLD patients had higher incidence of lifestyle-related diseases, worse indices of various metabolic abnormalities, and higher levels of non-invasive liver fibrosis scores than NAFLD patients in general population [31], none of these differences were observed in our study, and gender composition was the only significant difference (Table 2). It is expected that the patients enrolled in our study were at high risk of carcinogenesis since all of them had already developed HCC, which may explain the disappearance of the difference between the two groups.

In the study of MAFLD-HCC cases by diagnostic factors, all three factors (DM, overweight and MRA) included more than 60% of total cases, and therefore were considered to be essential in terms of distinguishing HCC patients (Fig. 3a). Huang et al. [48] reported that 28.5% of MAFLD patients in the general population were diagnosed with MAFLD with one factor, 50.2% with two factors, and 21.3% with all three factors combined. There were more cases with multiple factors than with only one also in our study including only HCC cases, and as many as 32% patients had all three factors, more than in the general population, which suggested an association between overlapping metabolic dysfunction and HCC development. Patients with overweight alone were younger and had more advanced liver fibrosis than other groups (Fig. 4a, c), and in addition, overweight patients accounted for the majority (88%, 64/73) of those under 70 years of age (Fig. 3b), suggesting that overweight may be an important factor contributing advanced liver fibrosis and the development of HCC in younger patients. Finally, we discussed the definition of overweight in diagnostic criteria for MAFLD. Kawaguchi et al. proposed setting BMI ≥ 25 kg/m^2^ in Japan because defining overweight as BMI ≥ 23 kg/m^2^could include muscular patients with no metabolic abnormalities and relatively low risk for intrahepatic or extrahepatic complications [49]. In our study, MAFLD-HCC still accounted for the majority of non-B, non-C HCC cases even after redefining overweight as BMI ≥ 25 kg/m^2^ (Fig. 5). This alleviation did not compromise the superiority of MAFLD, demonstrating the necessity of all three factors with complementary roles in the criteria, and the adequacy of BMI ≥ 25 kg/m^2^ as the definition of overweight also in terms of including HCC cases.

This study has two limitations. First, it was conducted at a single institution, and therefore, it is not certain whether similar results would be obtained in other geographic or ethnic cohorts. In addition, since this is a retrospective study, we cannot evaluate the difference in the risk of HCC development between MAFLD and NAFLD patients; prospective studies need to be conducted to more accurately assess the value of the disease concept of MAFLD.

In conclusion, MAFLD is a new disease concept that is superior to NAFLD in the inclusion of non-B, non-C HCC cases. In diagnosing MAFLD, we propose to define overweight as BMI ≥ 25 kg/m^2^ instead of ≥ 23 kg/m^2^. Further accumulation of cases and revision of the detailed criteria are needed so that it can be used to efficiently select patients with fatty liver who are at high risk of developing HCC.

## Supplementary Information

**Additional file 1: Table S1.**  Alcohol consumption for each group.

## Data Availability

The datasets used and analysed during the current study are available from the corresponding author on reasonable request.

## Abbreviations

NAFLD: Nonalcoholic fatty liver disease
MAFLD: Metabolic dysfunction-associated fatty liver disease
MRA: Metabolic risk abnormalities
BMI: Body mass index
HCC: Hepatocellular carcinoma
HOMA-IR: Homeostasis model assessment of insulin resistance
PNPLA3: Patatine-like phospholipase domain containing 3
