# Supplementary material for: Impact and usefulness of the transition to the new MAFLD classification for non-B, non-C HCC: a retrospective cohort study
Source: BMC Gastroenterol. 2023 Jun 28;23:222. doi: 10.1186/s12876-023-02851-y (PMC10303298; doi:10.1186/s12876-023-02851-y)
Supplement: Supplementary file 1 — Additional file 1: Table S1. Alcohol consumption for each group. [file 12876_2023_2851_MOESM1_ESM.docx]

**Table S1　Alcohol consumption for each group**

Data are expressed as total numbers and percentages.

|  | **none** | **Light drinkers** | **Moderate**  **drinkers** | **Heavy**  **drinkers** |
| --- | --- | --- | --- | --- |
| **MAFLD(+), NAFLD(-)** | 0 | 0 | 73 (57) | 55 (43) |
| **MAFLD(+), NAFLD(+)** | 80 (85) | 14 (15) | 0 | 0 |
| **MAFLD(-), NAFLD(+)** | 7 (100) | 0 | 0 | 0 |
| **MAFLD(-), NAFLD(-)** | 0 | 0 | 5 (63) | 3 (37) |
